# A bivariate Poisson regression to analyse impact of outlier women on correlation between female schooling and fertility in Malawi

**DOI:** 10.1186/s12905-024-02891-w

**Published:** 2024-01-20

**Authors:** Tsirizani Mwalimu Kaombe

**Affiliations:** Department of Mathematical Sciences, School of Natural and Applied Sciences, University of Malawi, Zomba, Malawi

**Keywords:** Female education, Fertility rate, Correlation, Sub-Saharan Africa, Survey data, Bivariate Poisson model, Outlier women

## Abstract

**Background:**

Women’s levels of education and fertility are commonly associated. In Sub-Saharan Africa, the pace of decreasing fertility rates varies greatly, and this is linked to women’s levels of education. However, this association may be influenced by unusual females who have uncommon measurements on both variables. Despite this, most studies that researched this association have only analysed the data descriptively, without taking into account the effect of potential outliers. This study aimed to examine the presence and impact of outlier women on the relationship between female education and fertility in Malawi, using regression methods.

**Methods:**

To analyse the correlation between women’s schooling and fertility and evaluate the effect of outliers on this relationship, a bivariate Poisson model was applied to three recent demographic and health surveys in Malawi. The R software version 4.3.0 was used for model fitting, outlier computations, and correlation analysis. The STATA version 12.0 was used for data cleaning.

**Results:**

The findings revealed a correlation of -0.68 to -0.61 between schooling and fertility over 15 years in Malawi. A few outlier women were identified, most of whom had either attended 0 or at least 9 years of schooling and had born either 0 or at least 5 children. The majority of the outliers were non-users of modern contraceptive methods and worked as domestic workers or were unemployed. Removing the outliers from the analysis led to marked changes in the fixed effects sizes and slight shifts in correlation, but not in the direction and significance of the estimates. The woman’s marital status, occupation, household wealth, age at first sex, and usage of modern contraceptives exhibited significant effects on education and fertility outcomes.

**Conclusion:**

There is a high negative correlation between female schooling and fertility in Malawi. Some outlier women were identified, they had either attended zero or at least nine years of schooling and had either born zero or at least five children. Most of them were non-users of modern contraceptives and domestic workers. Their impact on regression estimates was substantial, but minimal on correlation. Their identification highlights the need for policymakers to reconsider implementation strategies for modern contraceptive methods to make them more effective.

**Supplementary Information:**

The online version contains supplementary material available at 10.1186/s12905-024-02891-w.

## Introduction

The Total Fertility Rate (TFR) is the number of live births that a woman is expected to have in her lifetime [13]. This rate is especially high, around 5 children per woman, in sub-Saharan Africa (SSA) when compared to other regions of the world such as Europe, which has a TFR of about 2 per woman [10, 48, 74]. This difference in TFR can be attributed to various factors, including increased cases of early marriages, low education attainment, and lack of access to modern contraceptive methods [10, 50]. Women’s years of schooling, on the other hand, refers to the number of years spent in formal education during their lifetime [56]. This factor has a significant impact on their future participation in socio-economic activities. The level of a woman’s education is influenced by various factors, including early marriages, household wealth, parental education, religion, cultural norms, and division of labor within the home [1, 22, 30, 71]. Delayed marriage, for instance, is reported to contribute to increased years of schooling in females [67, 68].

There has been significant progress in women’s education in developing countries over the last 50 years [31]. This is likely due to increased awareness of human rights, including the right to education, that has come about with the adoption of democratic governments in these regions [38]. For instance, between 1970 and 2010, the average years of schooling for women in developing countries more than doubled from 2.99 to 7.2 [2]. Studies suggest a negative correlation between female education and fertility, meaning that as the number of years of education increases, the number of children born to a woman decreases, and vice versa [5, 7, 8, 18, 35, 46, 77]. This is mainly because pursuing higher education delays maternal age, while low education accelerates it [5]. However, this relationship varies across regions; it is stronger in the least developed countries than in developed nations, except for sub-Saharan Africa and Protestant Europe, where it is weak [36, 47, 54, 57].

There has been a decreasing trend in women’s fertility worldwide over the past 50 years, including in sub-Saharan Africa [14]. The decrease is largely due to improvements in women’s education attainment and family planning programmes, especially in developed nations. For example, it is reported that in Asia and Latin America, the total fertility rate (TFR) fell by about half between 1950 and the early 2000s [13–15, 43]. However, in sub-Saharan Africa, the pace of decline in TFR has been slow, steady, or even rising in some parts of the region, with an average TFR exceeding 5.1 births per woman in most parts of the region between 2005 and 2010 [14, 23, 26, 44]. This is mainly due to varying factors such as female marital ages, contraceptive use patterns, education attainment, and labor force participation by females in the region over time, among other factors [13–15, 23, 26, 32, 44, 54, 76]. The unstable trend of fertility outcomes in sub-Saharan Africa over time suggests the availability of some unusual fertility measurements in the region that are worth investigating. There have been reports of deteriorating human reproductive health in developed nations due to biological and environmental factors such as exposure to chemicals from fossil fuels [37, 49, 70].

The age at which a woman has her first child, her household’s wealth status, her parents’ education, birth intervals, age at first marriage, religion, and first sexual experience are all factors that can affect both her fertility and education [3, 4, 11, 27, 30, 45, 55, 64, 73]. Researchers often use a bivariate Poisson regression model to analyse the common determinants of both outcomes as they are often counts. This model can estimate the impact of these factors on both outcomes and the degree of correlation between them [6, 75]. However, some studies have only used descriptive statistical methods that do not thoroughly analyse the data, including outliers, and therefore fall short [48]. Despite the uneven trends of women’s fertility and the high variability of correlation between the total fertility rate and education in sub-Saharan Africa, little research examines the contribution of outlier females to the covariance of the two variables. This is mainly due to the lack of diagnostic statistics for nonlinear models such as the bivariate Poisson model [39, 42, 69]. This article applies diagnostic statistics to study outlier females and their impact on the correlation between fertility and education in Malawi, using data from three surveys conducted in 2004, 2010, and 2015-16.

The term “outlier women” refers to women whose fertility and education measurements do not fit the general pattern established by a bivariate Poisson model [39]. For instance, if the model indicates that women with low education tend to have more children, an outlier woman could have both fewer years of schooling and fewer children than expected. Outliers may be caused by either natural (i.e. genuine unusual measurements) or human (i.e. data handling errors) factors, and detecting them can improve the modelling process [42]. Outlier observations can have an exaggerated positive or negative impact on the effects of various covariates on the outcome(s) in the model, or no impact at all [40, 41].

It has been observed that the desired family sizes and female education in sub-Saharan Africa are major contributors to the average global TFR (total fertility rate) and women’s wellbeing [13, 14]. Therefore, when analysing the relationship between fertility and education in the region, it is important to take into account the outliers among females. This will help researchers avoid drawing false conclusions about the nature and strength of the association between the two variables. Such analysis will provide helpful insights for policymakers to develop appropriate national socio-economic policies concerning women’s health and livelihood in countries of the region, such as Malawi.

The paper is organised into the following sections: “Methods” section covers the data and statistical methods used, “Results” section outlines the results, “Discussion” section discusses the findings, and “Conclusion” section presents the conclusions.

## Methods

### Data

The study analysed data from the Malawi Demographic and Health Surveys (MDHSs) conducted in 2004, 2010, and 2015-16. The data included information about women aged 15 to 49 years and their education levels and fertility rates. The study used a regression method to measure the impact of outliers on the correlation between education attainment and fertility. The dependent variables used in the analysis were “education in single years” and “total children ever born,” while the covariates included variables like region of stay, woman’s religion, ethnicity, the current age of woman, age at first sex, woman’s occupation, place of residence, modern contraceptive use, marital status, and household wealth [3, 12, 14, 29, 30, 64, 73]. These variables were selected based on previous research. The data used in the study are publicly available, and the link to access the data is: https://dhsprogram.com/data/available-datasets.cfm.

Tables 1, 2 and 3 provide an overview of the three MDHS datasets. Across all characteristics of the studied women, the majority had attained 1-8 years of education, followed by 0 years and 9 years and above. However, for women with professional and formal occupations, the majority had 9 years and above education, followed by 1-8 years and 0 years. An exception to this trend was observed in the 2015-16 MDHS, where most women had 1-8 years of education, followed by 9 years and above, and then 0 years, as shown in Table 3. Regarding fertility, most women had given birth to 1 to 4 children, followed by 5 children and above, and then no child. This trend was consistent across all categories of women’s characteristics and years, except for unmarried women who had no children as the majority, followed by 1 to 4 children, and then 5 children and above. The median age at which a woman had her first sexual intercourse was 16 years, with a standard deviation of around 2.8 years, for women who belonged to the schooling bracket of 1-8 years and fertility range of 1 to 4 children, which was the majority of the studied women. The selected variables were useful in determining female education and fertility, as confirmed by the Chi-square test. The raw Spearman correlation coefficient between schooling and fertility variables was -0.39 in the 2004 and 2015 surveys and -0.41 in the 2010 survey. This indicates that there was a significantly high probability of a woman with more years of schooling having a smaller number of children ever born and vice versa. All these data summaries and cleaning were performed using the STATA package version 12. The STATA codes used are provided in Appendix 1.

**Table 1:** Distribution of schooling years and fertility by woman’s socio-demographic characteristics, 2004 MDHS

	Years of Schooling					Children ever born			
Characteristic	n (%)	0 (%)	1-8 (%)	9+ (%)	$\boldsymbol{\chi ^{2}}$ p-val	0 (%)	1-4 (%)	5+ (%)	$\boldsymbol{\chi ^{2}}$ p-val
Overall sample	11,698 (100)	2,823 (24.1)	7,262 (62.1)	1,613 (13.8)		2,400 (20.5)	6,134 (52.4)	3,164 (27.1)	
Ethinicity					$<0.0001$				$<0.0001$
Tumbuka/Tonga/other	3,618 (30.9)	527 (14.6)	2,403 (66.4)	688 (19.0)		804 (22.2)	1,861 (51.4)	953 (26.3)	
Lomwe/Yao/Sena	4,413 (37.7)	1,370 (31.0)	2,604 (59.0)	439 (10.0)		800 (18.1)	2,454 (55.6)	1,159 (26.3)	
Chewa/Nyanja	3,667 (31.4)	926 (25.3)	2,255 (61.5)	486 (13.3)		796 (21.7)	1,819 (49.6)	1,052 (28.7)	
Religion					$<0.0001$				$<0.0001$
None/other	127 (01.1)	63 (49.6)	55 (43.3)	9 (07.1)		14 (11.0)	65 (51.2)	48 (37.8)	
Muslim	1,816 (15.5)	753 (41.5)	953 (52.5)	110 (06.1)		293 (16.1)	1,023 (56.3)	500 (27.5)	
Christian	9,755 (83.4)	2,007 (20.6)	6,254 (64.1)	1,494 (15.3)		2,093 (21.5)	5,046 (51.7)	2,616 (26.8)	
Wealth status					$<0.0001$				$<0.0001$
Poor	4,409 (37.7)	1,536 (34.8)	2,710 (61.5)	163 (03.7)		728 (16.5)	2,355 (53.4)	1,326 (30.1)	
Middle	2,517 (21.5)	701 (27.9)	1,702 (67.6)	114 (04.5)		424 (16.8)	1,348 (53.6)	745 (29.6)	
Rich	4,772 (40.8)	586 (12.3)	2,850 (59.7)	1,336 (28.0)		1,248 (26.2)	2,431 (50.9)	1,093 (22.9)	
Marital status					$<0.0001$				$<0.0001$
Unmarried	1,902 (16.3)	60 (03.2)	1,232 (64.8)	610 (32.1)		1,705 (89.6)	194 (10.2)	3 (00.2)	
Married/cohabited	8,385 (71.7)	2,307 (27.5)	5,205 (62.1)	873 (10.4)		626 (07.5)	5,075 (60.5)	2,684 (32.0)	
Separated/other	1,411 (12.1)	456 (32.3)	825 (58.5)	130 (09.2)		69 (04.9)	865 (61.3)	477 (33.8)	
Occupation					$<0.0001$				$<0.0001$
Not working	4,733 (40.6)	973 (20.6)	2,968 (62.7)	792 (16.7)		1,412 (29.8)	2,334 (49.3)	987 (20.9)	
Domestic/Nonformal	5,430 (46.5)	1,522 (28.0)	3,421 (63.0)	487 (09.0)		605 (11.1)	3,022 (55.7)	1,803 (33.2)	
Professional/formal	1,504 (12.9)	323 (21.5)	854 (56.8)	327 (21.7)		376 (25.0)	760 (50.5)	368 (24.5)	
Contraceptive use					$<0.0001$				$<0.0001$
Non-user/other	9,164 (78.3)	2,258 (24.6)	5,699 (62.2)	1,207 (13.2)		2,347 (25.6)	4,617 (50.4)	2,200 (24.0)	
User	2,534 (21.7)	565 (22.3)	1,563 (61.7)	406 (16.0)		53 (02.1)	1,517 (59.9)	964 (38.0)	
Place of residence					$<0.0001$				$<0.0001$
Urban	1,640 (14.0)	128 (07.8)	858 (52.3)	654 (39.9)		472 (28.8)	895 (54.6)	273 (16.6)	
Rural	10,058 (86.0)	2,695 (26.8)	6,404 (63.7)	959 (09.5)		1,928 (19.2)	5,239 (52.1)	2,891 (28.7)	
Region					$<0.0001$				$<0.0001$
Northern	1,597 (13.7)	130 (08.1)	1,100 (68.9)	367 (23.0)		371 (23.2)	832 (52.1)	394 (24.7)	
Central	4,199 (35.9)	1,078 (25.7)	2,585 (61.6)	536 (12.8)		907 (21.6)	2,057 (49.0)	1,235 (29.4)	
Southern	5,902 (50.5)	1,615 (27.4)	3,577 (60.6)	710 (12.0)		1,122 (19.0)	3,245 (55.0)	1,535 (26.0)	
Median age (SD)		33 (8.77)	24 (8.86)	22 (6.76)		17 (4.80)	25 (6.57)	38 (6.34)	
Median age at 1st sex (SD)		16 (3.30)	16 (3.37)	18 (3.41)		18 (4.27)	16 (2.94)	16 (3.28)	
Raw correlation (pval)	-0.3918 ($<0.0001$)								

**Table 2:** Distribution of schooling years and fertility by woman’s socio-demographic characteristics, 2010 MDHS

	Years of Schooling					Children ever born			
Characteristic	n (%)	0 (%)	1-8 (%)	9+ (%)	$\boldsymbol{\chi ^{2}}$ p-val	0 (%)	1-4 (%)	5+ (%)	$\boldsymbol{\chi ^{2}}$ p-val
Overall sample	23,020 (100)	3,591 (15.6)	15,299 (66.5)	4,130 (17.9)		4,979 (21.6)	11,424 (49.6)	6,617 (28.7)	
Ethinicity					$<0.0001$				$<0.0001$
Tumbuka/Tonga/other	7,778 (33.8)	678 (08.7)	5,243 (67.4)	1,857 (23.9)		1,768 (22.7)	3,824 (49.2)	2,186 (28.1)	
Lomwe/Yao/Sena	7,443 (32.3)	1,500 (20.2)	4,767 (64.0)	1,176 (15.8)		1,513 (20.3)	3,875 (52.1)	2,055 (27.6)	
Chewa/Nyanja	7,799 (33.9)	1,413 (18.1)	5,289 (67.8)	1,097 (14.1)		1,698 (21.8)	3,725 (47.8)	2,376 (30.5)	
Religion					$<0.0001$				$<0.0001$
None/other	192 (00.8)	77 (40.1)	108 (56.3)	7 (03.6)		21 (10.9)	96 (50)	75 (39.1)	
Muslim	2,530 (11.0)	683 (27.0)	1,584 (62.6)	263 (10.4)		465 (18.4)	1,275 (50.4)	790 (31.2)	
Christian	20,298 (88.2)	2,831 (13.9)	13,607 (67.0)	3,860 (19.0)		4,493 (22.1)	10,053 (49.5)	5,752 (28.3)	
Wealth status					$<0.0001$				$<0.0001$
Poor	9,045 (39.3)	2,119 (23.4)	6,466 (71.5)	460 (05.1)		1,629 (18.0)	4,447 (49.2)	2,969 (32.8)	
Middle	4,721 (20.5)	759 (16.1)	3,467 (73.4)	495 (10.5)		887 (18.8)	2,471 (52.3)	1,363 (28.9)	
Rich	9,254 (40.2)	713 (07.7)	5,366 (58.0)	3,175 (34.3)		2,463 (26.6)	4,506 (48.7)	2,285 (24.7)	
Marital status					$<0.0001$				$<0.0001$
Unmarried	4,526 (19.7)	104 (02.3)	2,931 (64.8)	1,491 (32.9)		4,147 (91.6)	375 (08.3)	4 (00.1)	
Married/cohabited	15,445 (67.1)	2,889 (18.7)	10,357 (67.1)	2,199 (14.2)		739 (04.8)	9,137 (59.2)	5,569 (36.1)	
Separated/other	3,049 (13.2)	598 (19.6)	2,011 (66.0)	440 (14.4)		93 (03.1)	1,912 (62.7)	1,044 (34.2)	
Occupation					$<0.0001$				$<0.0001$
Not working	6,177 (26.8)	841 (13.6)	3,941 (63.8)	1,395 (22.6)		2,272 (36.8)	2,698 (43.7)	1,207 (19.5)	
Domestic/Nonformal	16,126 (70.1)	2,730 (16.9)	11,214 (69.5)	2,182 (13.5)		2,570 (15.9)	8,252 (51.2)	5,304 (32.9)	
Professional/formal	717 (03.1)	20 (02.8)	144 (20.1)	553 (77.1)		137 (19.1)	474 (66.1)	106 (14.8)	
Contraceptive use					0.490				$<0.0001$
Non-user/other	15,554 (67.6)	2,399 (15.4)	10,344 (66.5)	2,811 (18.1)		4,829 (31.0)	7,055 (45.4)	3,670 (23.6)	
User	7,466 (32.4)	1,192 (16.0)	4,955 (66.4)	1,319 (17.7)		150 (02.0)	4,369 (58.5)	2,947 (39.5)	
Place of residence					$<0.0001$				$<0.0001$
Urban	3,068 (13.3)	196 (06.4)	1,435 (46.8)	1,437 (46.8)		889 (29.0)	1,675 (54.6)	504 (16.4)	
Rural	19,952 (86.7)	3,395 (17.0)	13,864 (69.5)	2,693 (13.5)		4,090 (20.5)	9,749 (48.9)	6,113 (30.6)	
Region					$<0.0001$				$<0.0001$
Northern	4,189 (18.2)	150 (03.6)	3,043 (72.6)	996 (23.8)		908 (21.7)	2,070 (49.4)	1,211 (28.9)	
Central	7,862 (34.2)	1,383 (17.6)	5,266 (67.0)	1,213 (15.4)		1,801 (22.9)	3,648 (46.4)	2,413 (30.7)	
Southern	10,969 (47.6)	2,058 (18.8)	6,990 (63.7)	1,921 (17.5)		2,270 (20.7)	5,706 (52.0)	2,993 (27.3)	
Median age (SD)		36 (8.57)	26 (9.28)	24 (7.04)		17 (4.64)	26 (6.57)	38 (6.17)	
Median age at 1st sex (SD)		16 (3.32)	16 (2.96)	18 (3.18)		17 (3.97)	16 (2.72)	16 (2.87)	
Raw correlation (pval)	-0.4108 ($<0.0001$)								

**Table 3:** Distribution of schooling years and fertility by woman’s socio-demographic characteristics, 2015-16 MDHS

	Years of Schooling					Children ever born			
Characteristic	n (%)	0 (%)	1-8 (%)	9+ (%)	$\boldsymbol{\chi ^{2}}$ p-val	0 (%)	1-4 (%)	5+ (%)	$\boldsymbol{\chi ^{2}}$ p-val
Overall sample	24,562 (100)	2,988 (12.2)	15,355 (62.5)	6,219 (25.3)		5,574 (22.7)	13,162 (53.6)	5,826 (23.7)	
Ethinicity					$<0.0001$				$<0.0001$
Tumbuka/Tonga/other	7,741 (31.5)	527 (06.8)	4,736 (61.2)	2,478 (32.0)		1,771 (22.9)	4,185 (54.1)	1,785 (23.1)	
Lomwe/Yao/Sena	8,388 (34.2)	1,320 (15.7)	5,274 (62.9)	1,794 (21.4)		1,801 (21.5)	4,623 (55.1)	1,964 (23.4)	
Chewa/Nyanja	8,433 (34.3)	1,141 (13.5)	5,345 (63.4)	1,947 (23.1)		2,002 (23.7)	4,354 (51.6)	2,077 (24.6)	
Religion					$<0.0001$				$<0.0001$
None/other	151 (00.6)	58 (38.4)	69 (45.7)	24 (15.9)		23 (15.2)	79 (52.3)	49 (32.5)	
Muslim	2,726 (11.1)	614 (22.5)	1,731 (63.5)	381 (14.0)		530 (19.4)	1,476 (54.1)	720 (26.4)	
Christian	21,685 (88.3)	2,316 (10.7)	13,555 (62.5)	5,814 (26.8)		5,021 (23.2)	11,607 (53.5)	5,057 (23.3)	
Wealth status					$<0.0001$				$<0.0001$
Poor	8,708 (35.5)	1,706 (19.6)	6,338 (72.8)	664 (07.6)		1,491 (17.1)	4,840 (55.6)	2,377 (27.3)	
Middle	4,508 (18.4)	599 (13.3)	3,270 (72.5)	639 (14.2)		928 (20.6)	2,331 (51.7)	1,249 (27.7)	
Rich	11,346 (46.2)	683 (06.0)	5,747 (50.7)	4,916 (43.3)		3,155 (27.8)	5,991 (52.8)	2,200 (19.4)	
Marital status					$<0.0001$				$<0.0001$
Unmarried	5,326 (21.7)	103 (01.9)	3,074 (57.7)	2,149 (40.3)		4,595 (86.3)	710 (13.3)	21 (00.4)	
Married/cohabited	15,952 (64.9)	2,283 (14.3)	10,244 (64.2)	3,425 (21.5)		886 (05.6)	10,336 (64.8)	4,730 (29.7)	
Separated/other	3,284 (13.4)	602 (18.3)	2,037 (62.0)	645 (19.6)		93 (02.8)	2,116 (64.4)	1,075 (32.7)	
Occupation					$<0.0001$				$<0.0001$
Not working	8,422 (34.3)	805 (09.6)	5,261 (62.5)	2,356 (28.0)		3,209 (38.1)	3,906 (46.4)	1,307 (15.5)	
Domestic/Nonformal	14,093 (57.4)	2,095 (14.9)	9,399 (66.7)	2,599 (18.4)		2,059 (14.6)	7,862 (55.8)	4,172 (29.6)	
Professional/formal	2,047 (08.3)	88 (04.3)	695 (34.0)	1,264 (61.7)		306 (14.9)	1,394 (68.1)	347 (17.0)	
Contraceptive use					$<0.0001$				$<0.0001$
Non-user/other	13,574 (55.3)	1,591 (11.7)	8,279 (61.0)	3,704 (27.3)		5,248 (38.7)	5,934 (43.7)	2,392 (17.6)	
User	10,988 (44.7)	1,397 (10.3)	7,076 (52.1)	2,515 (18.5)		326 (02.4)	7,228 (53.2)	3,434 (25.3)	
Place of residence					$<0.0001$				$<0.0001$
Urban	5,247 (21.4)	227 (04.3)	2,215 (42.2)	2,805 (53.5)		1,521 (29.0)	3,082 (58.7)	644 (12.3)	
Rural	19,315 (78.6)	2,761 (14.3)	13,140 (68.0)	3,414 (17.7)		4,053 (21.0)	10,080 (52.2)	5,182 (26.8)	
Region					$<0.0001$				$<0.0001$
Northern	4,803 (19.6)	196 (04.1)	3,015 (62.8)	1,592 (33.1)		1,090 (22.7)	2,598 (54.1)	1,115 (23.2)	
Central	8,417 (34.3)	1,104 (13.1)	5,272 (62.6)	2,041 (24.2)		2,038 (24.2)	4,362 (51.8)	2,017 (24.0)	
Southern	11,342 (46.2)	1,688 (14.9)	7,068 (62.3)	2,586 (22.8)		2,446 (21.6)	6,202 (54.7)	2,694 (23.8)	
Median age (SD)		37 (8.82)	26 (9.28)	25 (7.57)		17 (4.)	26 (6.93)	39 (5.74)	
Median age at 1st sex (SD)		15 (2.72)	16 (2.61)	18 (3.10)		17 (3.57)	16 (2.60)	16 (2.45)	
Raw correlation (pval)	-0.3917 ($<0.0001$)								

### Bivariate Poisson regression model

Suppose that $Y_{i1}$ represents the total number of years of schooling for a woman and $Y_{i2}$ the total number of children she has ever had, where $i = 1, 2, ..., n$. Let $y_{i1}$ and $y_{i2}$ be the actual observed paired counts for each woman. The average number of years of schooling for a woman in the country, denoted by $\theta _{1}=E(Y_{i1})$ can be calculated. Similarly, $\theta _{2}=E(Y_{i2})$ is the average number of children ever born by a woman in the country. If $\theta _{3}=cov(Y_{i1}, Y_{i2})$ is the covariance between the two variables and $\theta _{1}=E(Y_{i1})=Var(Y_{i1})$ while $\theta _{2}=E(Y_{i2})=Var(Y_{i2})$, then the joint distribution of $Y_{i1}$ and $Y_{i2}$ can be expressed using a bivariate Poisson random variable [6, 75]. The distribution has a probability mass function (pmf) given by:

1 $$\begin{aligned} f(y_{i1},y_{i2}|\theta _{1},\theta _{2},\theta _{3}){} & {} =exp(-\theta _{1}-\theta _{2}-\theta _{3})\frac{\theta _{1}^{y_{i1}}}{y_{i1}!} \frac{\theta _{2}^{y_{i2}}}{y_{i2}!}\sum \limits _{k=0}^{min(y_{i1},y_{i2})}k!\left( \frac{\theta _{3}}{\theta _{1}\theta _{2}}\right) ^{k}\times \left( {\begin{array}{c}y_{i1}\\ k\end{array}}\right) \times \left( {\begin{array}{c}y_{i2}\\ k\end{array}}\right) \nonumber \\{} & {} =exp\left[ y_{i1}log\theta _{1}+y_{i2}log\theta _{2}-\theta _{1}-\theta _{2}-\theta _{3}+log \left( \sum \limits _{k=0}^{min(y_{i1},y_{i2})}\frac{\theta _{1}^{-k}\theta _{2}^{-k}\theta _{3}^{k}}{k!(y_{i1}-k)!(y_{i2}-k)!}\right) \right] , \end{aligned}$$

where $y_{i1}, y_{i2}, \theta _{1}, \theta _{2} \ge 0$, and $\theta _{3} \in R$. The second line of the bivariate Poisson pmf in Eq. (1) represents the exponential family form of the distribution in the first line. This is obtained by exponentiating the logarithm of the expression in the first line and simplifying the terms.

Equation (1) reveals that the probability distribution of a bivariate Poisson random variable is in canonical form and has two natural parameters, namely $log\theta _{1}$ and $log\theta _{2}$. Therefore, the bivariate Poisson regression model needs to be defined with two link functions for these parameters, as well as a correlation term, to determine the effects of explanatory variables on the paired outcome $(Y_{i1}, Y_{i2})$ [42]. If ${\textbf {x}}^{T}_{ir}=(1,x_{i1},x_{i2},...,x_{ip})$ represents a vector of covariate values observed on the *i*-th woman, where $x_{i0}=1$, then the bivariate Poisson regression model can be expressed as simultaneous equations given by:

2 $$\begin{aligned} Y_{ij}{} & {} =\theta _{ij}({\textbf {x}}) + \epsilon _{ij}, \hspace{10pt} i = 1,2,...,n; j=1,2, \nonumber \\ \theta _{i3}{} & {} =q({\textbf {x}}), \end{aligned}$$

where $Y_{ij} = (Y_{i1}, Y_{i2})$ are the two response variables, $\theta _{ij}({\textbf {x}})=(\theta _{i1}({\textbf {x}}),\theta _{i2}({\textbf {x}}))$ the marginal conditional expected counts for $Y_{i1}$ and $Y_{i2}$ given the covariates *X*, respectively. The term $\theta _{i3}$ is the dependence measure between $Y_{i1}$ and $Y_{i2}$ estimated from the model. The marginal error term for the model is represented by $\epsilon _{ij}$. Assuming that $\epsilon _{ij}$ has mean zero, then the conditional mean of the marginal responses $Y_{ij}$ is $E(Y_{ij}|X)=\theta _{ij}({\textbf {x}})$, which is the part of the model that links or relates with the explanatory variables [42].

Therefore, the bivariate Poisson model in Eq. (2) can be further defined in terms of the two link functions in the pmf given in Eq. (1) and the correlation term, as follows:

3 $$\begin{aligned} log[\theta _{i1}({\textbf {x}})]{} & {} ={\textbf {x}}_{1ir}^{T}\beta , \nonumber \\ log[\theta _{i2}({\textbf {x}})]{} & {} ={\textbf {x}}_{2ir}^{T}\beta , \nonumber \\ \theta _{i3}{} & {} =q({\textbf {x}}), \end{aligned}$$

where $\beta =(\beta _0,\beta _1,...,\beta _p)^{T}$ is a column vector of regression coefficients and ${\textbf {x}}_{ir}^{T}=(1,x_{i1},x_{i2},...,x_{ip})$ is a row vector of covariates observed on the *i*-th woman, $r = 1,2,3,...,p$. The linear operators associated with the first and second marginal models are represented by ${\textbf {x}}_{1ir}^{T}\beta$ and ${\textbf {x}}_{2ir}^{T}\beta$, respectively. The quantity *q*(.) is the correlation function, that is estimated from the model’s data [42]. Since there are two natural parameters for the bivariate Poisson distribution, the covariance term $\theta _{i3}$ is considered a nuisance parameter, and its estimation in the model in Eq. (3) is done after the first two marginal models have been estimated [40, 72]. The dependence term, denoted by $\theta _{i3}({\textbf {x}})$, is often reported as the correlation coefficient between the two outcomes, since the units for $Y_{i1}$ and $Y_{i2}$ may be different. This coefficient is dimensionless, as opposed to covariance [72]. The bivariate Poisson model can be presented as either a parallel or non-exchangeable model, where the effects of the covariates on marginal outcomes are unique to each outcome. Alternatively, the effects of the covariates can be restricted to be common for both marginal outcomes, resulting in the exchangeable model [42]. In this study, the non-exchangeable (parallel) bivariate Poisson model was used to estimate the separate effects of the covariates on marginal models and then estimate the marginal as well as overall outlier females to the bivariate model. Recent research has shown that outliers to the marginal bivariate models can be candidates for joint outliers to the entire bivariate model [42]. Therefore, using the non-exchangeable (parallel) bivariate Poisson model allowed us to estimate separate effects of the covariates on the marginal models and then estimate the marginal as well as overall outlier females to the bivariate model.

The likelihood function for the model in Eq. (3) is obtained by multiplying the probabilities of joint counts of the two outcomes for individual women in Eq. (1) as follows:

4 $$\begin{aligned} L(\theta ){} & {} =\prod _{i=1}^{n}exp\left[ y_{i1}ln\theta _{1}+y_{i2}ln\theta _{2}-\theta _{1}-\theta _{2}-\theta _{3}+ln \left( \sum \limits _{k=0}^{min(y_{i1},y_{i2})}\frac{\theta _{1}^{-k}\theta _{2}^{-k}\theta _{3}^{k}}{k!(y_{i1}-k)!(y_{i2}-k)!}\right) \right] \nonumber \\{} & {} =exp\left[ \sum \limits _{i=1}^{n} \left( y_{i1}ln\theta _{1}+y_{i2}ln\theta _{2}-\theta _{1}-\theta _{2}-\theta _{3}+ln \left( \sum \limits _{k=0}^{min(y_{i1},y_{i2})}\frac{\theta _{1}^{-k}\theta _{2}^{-k}\theta _{3}^{k}}{k!(y_{i1}-k)!(y_{i2}-k)!}\right) \right) \right] . \end{aligned}$$

The log-likelihood function is obtained by taking the natural logarithm of the likelihood function in Eq. (4) and is expressed as a function of the model parameters $\theta = (\theta _{1},\theta _{2},\theta _{3})$ as follows:

5 $$\begin{aligned} l(\theta ){} & {} =\sum \limits _{i=1}^{n} \left[ y_{i1}ln\theta _{1}+y_{i2}ln\theta _{2}-\theta _{1}-\theta _{2}-\theta _{3}+ln \left( \sum \limits _{k=0}^{min(y_{i1},y_{i2})}\frac{\theta _{1}^{-k}\theta _{2}^{-k}\theta _{3}^{k}}{k!(y_{i1}-k)!(y_{i2}-k)!}\right) \right] \nonumber \\{} & {} =\sum \limits _{i=1}^{n} \left[ y_{i1}{} {\textbf {x}}_{1ir}^{T}\beta +y_{i2}{} {\textbf {x}}_{2ir}^{T}\beta -exp({\textbf {x}}_{1ir}^{T}\beta )-exp({\textbf {x}}_{1ir}^{T}\beta )-q({\textbf {x}})+ln \left( \sum \limits _{k=0}^{min(y_{i1},y_{i2})}\frac{(exp({\textbf {x}}_{1ir}^{T}\beta ))^{-k}(exp({\textbf {x}}_{2ir}^{T}\beta ))^{-k}q({\textbf {x}})^{k}}{k!(y_{i1}-k)!(y_{i2}-k)!}\right) \right] . \end{aligned}$$

To find the score vector for the model, the partial derivatives of the log-likelihood function in Eq. (5) are taken with respect to $\beta$ as follows:

6 $$\begin{aligned} \frac{\partial l(\beta )}{\partial \beta _{{\textbf {x}}1}}{} & {} =\sum \limits _{i=1}^{n} \left[ {\textbf {x}}_{1ir}^{T}\left( y_{i1}-exp({\textbf {x}}_{1ir}^{T}\beta )\right) -\frac{\sum _{k=0}^{min(y_{i1},y_{i2})}\frac{{\textbf {x}}_{1ir}^{T}(exp({\textbf {x}}_{1ir}^{T}\beta ))^{-k}(exp({\textbf {x}}_{2ir}^{T}\beta ))^{-k}q({\textbf {x}})^{k}}{(k-1)!(y_{i1}-k)!(y_{i2}-k)!}}{\sum _{k=0}^{min(y_{i1},y_{i2})}\frac{(exp({\textbf {x}}_{1ir}^{T}\beta ))^{-k}(exp({\textbf {x}}_{2ir}^{T}\beta ))^{-k}q({\textbf {x}})^{k}}{k!(y_{i1}-k)!(y_{i2}-k)!}}\right] \nonumber \\ \frac{\partial l(\beta )}{\partial \beta _{{\textbf {x}}2}}{} & {} =\sum \limits _{i=1}^{n} \left[ {\textbf {x}}_{2ir}^{T}\left( y_{i2}-exp({\textbf {x}}_{2ir}^{T}\beta )\right) -\frac{\sum _{k=0}^{min(y_{i1},y_{i2})}\frac{{\textbf {x}}_{2ir}^{T}(exp({\textbf {x}}_{2ir}^{T}\beta ))^{-k}(exp({\textbf {x}}_{1ir}^{T}\beta ))^{-k}q({\textbf {x}})^{k}}{(k-1)!(y_{i1}-k)!(y_{i2}-k)!}}{\sum _{k=0}^{min(y_{i1},y_{i2})}\frac{(exp({\textbf {x}}_{1ir}^{T}\beta ))^{-k}(exp({\textbf {x}}_{2ir}^{T}\beta ))^{-k}q({\textbf {x}})^{k}}{k!(y_{i1}-k)!(y_{i2}-k)!}}\right] , \end{aligned}$$

where $\beta _{{\textbf {x}}1}$ and $\beta _{{\textbf {x}}2}$ are regression parameter vectors associated with marginal models 1 and 2 in Eq. (3), respectively.

The process is finalised by equating the score vectors in Eq. (6) to zero, after which the parameter values are calculated numerically because the obtained equations are not in closed form. The computaions were implemented using the R package VGAMdata, which is designed to analyse vector generalised linear and additive models [78]. The maximum likelihood estimate, denoted as $\hat{\beta }$, was understood as the change in the logarithm of the expected number of years of schooling or TFR that corresponded to a unit change in the value of a covariate. However, the R package VGAMdata had some limitations with respect to processing the correlation estimate $\hat{\theta }_{i3}$ in the model in Eq. (3). Therefore, the Spearman’s rank correlation was used to post-estimate it, taking into account the skewed nature of the data, as outlined in [61]. The correlation measure is expressed as follows:

7 $$\begin{aligned} corr(\hat{Y}_{1},\hat{Y}_{2})=1-\frac{6\sum _{i=1}^{n} d^{2}_{i}}{n(n^{2}-1)}, \end{aligned}$$

where $d_{i}$ was the distance between the rank of a fitted marginal schooling outcome, $\hat{\theta }_{i1}({\textbf {x}})$ and the rank of a fitted marginal fertility outcome, $\hat{\theta }_{i2}({\textbf {x}})$ associated with the *i*-th woman, and *n* was the sample size.

A correlation value of zero meant that there was no linear relationship between a woman’s years of schooling and the number of children she had. A negative correlation indicated that women with higher levels of education tended to have fewer children, while those with lower levels of education tended to have more children. A positive correlation indicated the opposite [61]. To illustrate this correlation, scatter plots were used. The analysis was conducted using the R software version 4.3.0 and relevant packages. The best model was selected using the Akaike information criterion (AIC), given by $-2l(\theta ) + 2p$, which takes into account the number of regression parameters *p* in the model. Initially, a model with all covariates was fitted to the data, and its AIC value was observed. Then, covariates with large *p*-values were excluded, and the AIC was observed again. The model with the lowest AIC was considered the best model and used for subsequent computations [51].

### Analysis of outlier women to the bivariate Poisson model

One of the simplest statistics for detecting outlier observations in a generalised linear model is the deviance residual. In the case of bivariate models, this can be done by first calculating marginal deviance residuals for each marginal model and then averaging the obtained marginal residuals [42]. For the bivariate Poisson regression model, a marginal deviance residual is defined as:

8 $$\begin{aligned} d_{ij} = sgn(y_{ij}-\hat{\theta }_{ij}({\textbf {x}})) \left[ 2\left[ y_{ij}log \left( \frac{y_{ij}+\delta }{\hat{\theta }_{ij}({\textbf {x}})}\right) -(y_{ij}-\hat{\theta }_{ij}({\textbf {x}}))\right] \right] ^{1/2}, \end{aligned}$$

where $y_{ij}$ is *i*-th observation for the *j*-th outcome, $\hat{\theta }_{ij}({\textbf {x}})=exp({\textbf {x}}_{jir}^{T}\hat{\beta })$ is the marginal fitted count outcome, and *sgn*(.) is the signum function of the residual $y_{ij}-\hat{\theta }_{ij}({\textbf {x}})$, which takes the value of $+1$ if the residual was greater than zero, $-1$ when the residual was less than zero, and 0 if the residual was zero, $i=1,2,...,n$, and $j=1,2$ [42]. The term $\delta = 0.000001$ was an arbitrarily chosen smoothing constant that ensured convergence of the residual to real solutions for all values of women’s schooling and fertility. Adapting the concepts of kriging in spatial statistics and time-series analysis [20] and white noise smoothing in non-parametric regression [33], the term $\delta = 0.000001$ in Eq. (8) ensured that the residual does not converge to negative infinity for zero measurements of fertility or schooling but to analytic values while maintaining the variances of the two Poisson random variables. The marginal deviance residual in Eq. (8) has an assumed normal probability distribution with mean zero, hence the values at its extreme ends are indicative of outliers to the marginal fitted model [42].

The overall outlier statistic for the bivariate Poisson model was obtained by computing the average of the marginal deviance residuals found in Eq. (8) [42, 69] given by:

9 $$\begin{aligned} d^{*}_{i}=\frac{1}{2}(d_{i1}+d_{i2}), \end{aligned}$$

where the variables $d_{i1}$ and $d_{i2}$ represent the marginal deviance residuals for the schooling and fertility outcomes. The residual statistic in Eq. (9) is assumed to follow a normal probability distribution with a mean of zero. As such, its large absolute values correspond to the outlier observations to the fitted bivariate Poisson model [42]. Outlier observations to the fitted bivariate Poisson model were identified by plotting the deviance residual in Eq. (9) against individual women identification numbers using cutoffs of $\pm 1.96$ and $\pm 2.58$. Once the outliers were identified, they were removed from the dataset. The bivariate Poisson model was then refitted to the remaining sample. The fitted values and correlation estimate were recomputed from the new fitted model to observe the change in correlation value between the schooling and fertility variables. This process was carried out for all three MDHS data sets, as described in “Data” section. These calculations were done using R software version 4.3.0. All the R codes used to implement the methods described in this section are provided in Appendix 2.

## Results

### Bivariate Poisson regression model estimates

The data in Table 4 shows the results of the bivariate Poisson model’s maximum likelihood estimates. This model estimated the impact of women’s factors on the joint outcome of schooling years and fertility, using the three MDHS data sets. The results indicated that without taking into consideration the women’s characteristics, the logarithm of the expected number of years of schooling would increase by a factor of 1.5 in 2004 and 2010, and 1.4 in 2015-16. At the same time, the logarithm of the expected number of children born by a woman would decrease by 2.7 in 2004 and 2010, and by 2.2 in 2015-16. Furthermore, the results indicated that the logarithm of the expected number of years of schooling was significantly higher in Muslim and Christian women compared to non-religious women, in women from middle and rich households compared to poor households, in women who got separated or divorced from their husbands compared to those unmarried, in women with professional and formal occupations compared to those not working, in women who used modern contraceptive methods compared to non-users or others, and in women who had older age at first sex. On the other hand, the log-mean number of years of schooling was lower in *Lomwe*, *Yao*, *Sena*, *Chewa* and *Nyanja* tribes compared to *Tumbuka*, *Tonga*, *Ngoni*, and other related tribes. It was also lower in married women compared to unmarried women, in women with domestic and non-formal occupations compared to women who were not working, in women from rural locations compared to urban locations, in women from central and southern regions compared to the northern region, and in older women.

The results presented in Table 4 suggest that the average number of children ever born by a woman is higher among married and separated/divorced women compared to unmarried women. Similarly, women with domestic and nonformal occupations, those who use modern contraceptives, those from rural areas, and older women tended to have a higher number of children. Conversely, the expected number of children ever born by a woman was lower for women from middle and rich households, those with professional and formal occupations, and those who had their first sexual encounter at an older age. The study found that the effects of region, religion, and ethnicity on women’s fertility were not statistically significant. Furthermore, the study also computed the correlation between female schooling and fertility using the bivariate Poisson model. The results showed a negative correlation between schooling and fertility, with women who had more years of schooling having fewer children ever born. The estimated Spearman rank correlation values for the years 2004, 2010, and 2015-16 were -0.627, -0.681, and -0.621, respectively. These values were significantly different from zero and about double the raw correlation estimates given in “Data” section for all three surveys, indicating that the correlation estimates were strengthened by considering various women characteristics in the computation. The study did not drop any covariates to observe the change in AIC values since all the studied variables had significant effects on either schooling or fertility, although the AIC values were also computed and presented in Table 4.

**Table 4:** Effects of women socio-demographic characteristics on years of schooling and fertility outcomes upon fitting bivariate Poisson model to full MDHS data

	2004MDHS ($\boldsymbol{n=11,698}$)		2010 MDHS ($\boldsymbol{n=23,020}$)		2015-16 MDHS ($\boldsymbol{n=24,562}$)	
	Schooling	Fertility	Schooling	Fertility	Schooling	Fertility
Variable	Log-Mean (pv)	Log-Mean (pv)	Log-Mean (pv)	Log-Mean (pv)	Log-Mean (pv)	Log-Mean (pv)
Intercept	1.47 ($<0.0001$)	-2.74 ($<0.0001$)	1.48 ($<0.0001$)	-2.74 ($<0.0001$)	1.42 ($<0.0001$)	-2.17 ($<0.0001$)
Ethnicity						
Tumbuka/Tonga/ot$^\mathrm{a}$						
Lomwe/Yao/Sena	-0.132 ($<0.0001$)	0.001 (0.963)	-0.094 ($<0.0001$)	0.001 (0.935)	-0.058 ($<0.0001$)	-0.013 (0.300)
Chewa/Nyanja	-0.099 ($<0.0001$)	0.050 (0.002)	-0.105 ($<0.0001$)	0.024 (0.0327)	-0.087 ($<0.0001$)	0.019 (0.0834)
Religion						
None/Other$^\mathrm{a}$						
Muslim	0.147 (0.014)	0.036 (0.452)	-0.183 ($<0.0001$)	0.014 (0.295)	0.153 (0.0005)	0.110 (0.0159)
Christian	0.448 ($<0.0001$)	0.002 (0.963)	-0.186 ($<0.0001$)	-0.021 (0.115)	0.347 ($<0.0001$)	0.025 (0.573)
Wealth						
Poor$^\mathrm{a}$						
Middle	0.176 ($<0.0001$)	-0.009 (0.523)	0.220 ($<0.0001$)	-0.057 ($<0.0001$)	0.180 ($<0.0001$)	-0.040 (0.0002)
Rich	0.563 ($<0.0001$)	-0.079 ($<0.0001$)	0.466 ($<0.0001$)	-0.115 ($<0.0001$)	0.416 ($<0.0001$)	-0.112 ($<0.0001$)
Marital status						
Unmarried$^\mathrm{a}$						
Married/cohabited	-0.062 ($<0.0001$)	2.47 ($<0.0001$)	-0.042 ($<0.0001$)	2.71 ($<0.0001$)	-0.020 (0.0112)	1.98 ($<0.0001$)
Separated/other	0.060 (0.0015)	2.34 ($<0.0001$)	0.057 ($<0.0001$)	2.55 ($<0.0001$)	0.004 (0.706)	1.902 ($<0.0001$)
Occupation						
Not working$^\mathrm{a}$						
Domestic/informal	-0.027 (0.0068)	0.031 (0.0088)	-0.006 (0.376)	0.033 (0.0004)	-0.0003 (0.956)	0.036 ($<0.0001$)
Professional/formal	0.122 ($<0.0001$)	0.009 (0.611)	0.507 ($<0.0001$)	-0.198 ($<0.0001$)	0.334 ($<0.0001$)	-0.080 ($<0.0001$)
Contraceptive use						
Non-user/other$^\mathrm{a}$						
User	0.144 ($<0.0001$)	0.183 ($<0.0001$)	0.079 ($<0.0001$)	0.159 ($<0.0001$)	0.044 ($<0.0001$)	0.188 ($<0.0001$)
Place of residence						
Urban$^\mathrm{a}$						
Rural	-0.248 ($<0.0001$)	0.126 ($<0.0001$)	-0.185 ($<0.0001$)	0.135 ($<0.0001$)	-0.185 ($<0.0001$)	0.145 ($<0.0001$)
Region						
Northern$^\mathrm{a}$						
Central	-0.166 ($<0.0001$)	0.019 (0.351)	-0.183 ($<0.0001$)	0.014 (0.295)	-0.087 ($<0.0001$)	-0.023 (0.0727)
Southern	-0.153 ($<0.0001$)	-0.039 (0.0496)	-0.186 ($<0.0001$)	-0.021 (0.115)	-0.107 ($<0.0001$)	-0.019 (0.148)
Current age	-0.027 ($<0.0001$)	0.058 ($<0.0001$)	-0.025 ($<0.0001$)	0.055 ($<0.0001$)	-0.021 ($<0.0001$)	0.058 ($<0.0001$)
Age at 1st sex	0.026 ($<0.0001$)	-0.026 ($<0.0001$)	0.028 ($<0.0001$)	-0.032 ($<0.0001$)	0.036 ($<0.0001$)	-0.041 ($<0.0001$)
Correlation (pv)	-0.627 ($<0.0001$)		-0.681 ($<0.0001$)		-0.621 ($<0.0001$)	
AIC	100,903.14		192,796.76		203,069.20	

$^\mathrm{a}$ = reference category, pv = *p*-value

### Outlier observations to the fitted bivariate Poisson model

**Fig. 1:**
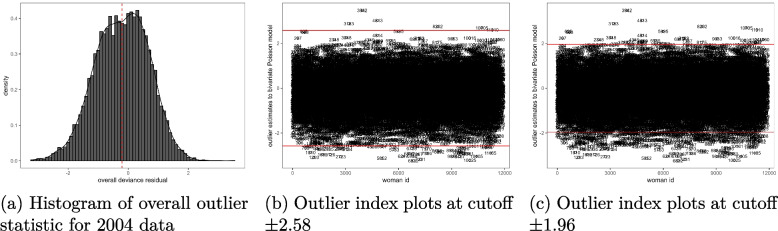
Histogram and index plots of the outlier statistic for a bivariate schooling and fertility Poisson model, 2004 MDHS data. Source: Researcher

**Fig. 2:**
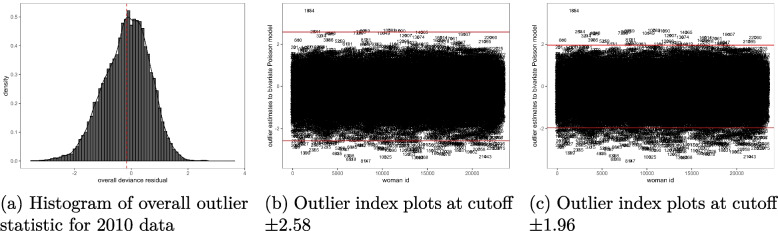
Histogram and index plots of the outlier statistic for a bivariate schooling and fertility Poisson model, 2010 MDHS data. Source: Researcher

**Fig. 3:**
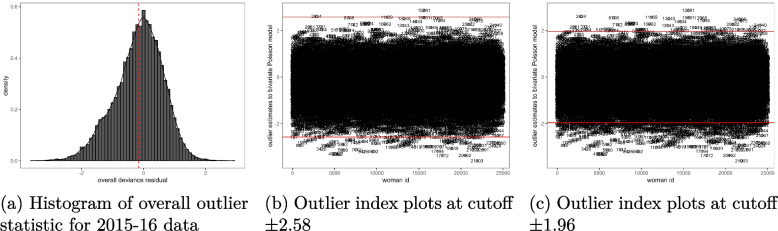
Histogram and index plots of the outlier statistic for a bivariate schooling and fertility Poisson model, 2015-16 MDHS data. Source: Researcher

The Figs. 1(a)-3(c) provide the results for outlier observations. It is shown in the histograms given in Figs. 1(a), 2(a) and 3(a) that the applied outlier statistic for the bivariate Poisson model had an approximate standard normal probability distribution. Therefore, the cutoffs suggested in “Analysis of outlier women to the bivariate Poisson model” section for outlier analysis were applied. At a threshold of $\pm 2.58$, the outlier residual detected 56 outlying observations in the 2004 data model, as shown in Fig. 1(b), and 329 were detected at $\pm 1.96$, as seen in Fig. 1(c). For the 2010 data, the residual identified 100 outliers using the $\pm 2.58$ threshold, as illustrated in Fig. 2(b), and 449 outliers at cutoff $\pm 1.96$, as shown in Fig. 2(c). In the case of the 2015-16 MDHS data model, 78 outliers were detected at the $\pm 2.58$ cutoff, see Fig. 3(b), and 490 at $\pm 1.96$ cutoff, Fig. 3(c). Overall, the majority of observations were well-fitted by the bivariate Poisson model across all the data sets, suggesting that the model was appropriate for these data.

In each data set, most of the identified outliers were cases where a subject’s measurement was over-predicted by the bivariate Poisson model. These were subjects with residual values below -2.5 at $\pm 2.58$ cutoff in Figs. 1(b), 2(b), and 3(b), and less than -1.96 for cutoff $\pm 1.96$ in Figs. 1(c), 2(c), and 3(c). This means that these observations had smaller actual measurements on schooling and fertility than those predicted by the model. On the other hand, the observations that were under-predicted by the model were few - those cases with a residual value above 2.58 using cutoff $\pm 2.58$ in Figs. 1(b), 2(b), and 3(b) and greater than 1.96 using cutoff $\pm 1.96$ in Figs. 1(c), 2(c), and 3(c). This implies that their actual measurements on fertility and schooling were larger than the ones estimated by the model. These results can also be confirmed from the dotted mean line of the overall deviance residual in Figs. 1(a), 2(a), and 3(a) that shifted to the left of zero, suggesting the presence of more outliers to the left the residual’s central point of zero that to its right.

While analysing the main data files, it was noticed that a significant number of women who attended at least nine years of schooling and had given birth to at least five children were under-predicted by the model. On the other hand, a large proportion of those who were over-predicted by the model had attended zero years of schooling and had not given birth to any children in their lifetime. In both groups of outliers, it was observed that the majority of them were non-users of modern contraceptive methods and worked as domestic workers or had non-formal jobs. Additionally, it was discovered that the outliers had a similar correlation structure as the well-fitted data when analysed separately.

### Effects of outliers on the bivariate Poisson model fixed-effect estimates and correlation

Table 5 presents the estimates for the parameters and correlation that were obtained from the models after excluding the outliers from the datasets, based on a cutoff value of $\pm 2.58$ of the deviance residual. The results indicate that the impact of ethnicity, place of residence, household wealth, and religion on schooling outcomes slightly increased after deleting the outliers. However, the effects of marital status and the use of modern contraceptive methods on schooling have slightly decreased. There was no change in the effect of age at first sex on both schooling and fertility. Additionally, the effects of household wealth and marital status on a woman’s fertility outcome increased. As before, religion, region, and ethnicity had no significant effects on a woman’s fertility. The correlation estimates have slightly decreased across all three datasets. Additionally, the AIC values for the models had decreased, indicating a better fit upon dropping the outliers.

**Table 5:** Effects of women socio-demographic characteristics on years of schooling and fertility outcomes upon fitting bivariate Poisson model to the MDHS data sets without outlier observations beyond cutoff $\pm 2.58$ of deviance residual

	2004MDHS ($\boldsymbol{n=11,642}$)		2010 MDHS ($\boldsymbol{n=22,920}$)		2015-16 MDHS ($\boldsymbol{n=24,484}$)	
	Schooling	Fertility	Schooling	Fertility	Schooling	Fertility
Variable	Log-Mean (pv)	Log-Mean (pv)	Log-Mean (pv)	Log-Mean (pv)	Log-Mean (pv)	Log-Mean (pv)
Intercept	1.45 ($<0.0001$)	-2.83 ($<0.0001$)	1.47 ($<0.0001$)	-2.81 ($<0.0001$)	1.41 ($<0.0001$)	-2.19 ($<0.0001$)
Ethnicity						
Tumbuka/Tonga/ot$^\mathrm{a}$						
Lomwe/Yao/Sena	-0.131 ($<0.0001$)	0.000 (0.997)	-0.095 ($<0.0001$)	-0.003 (0.8059)	-0.058 ($<0.0001$)	-0.009 (0.4713)
Chewa/Nyanja	-0.098 ($<0.0001$)	0.046 (0.004)	-0.106 ($<0.0001$)	0.021 (0.0612)	-0.087 ($<0.0001$)	0.019 (0.0946)
Religion						
None/Other$^\mathrm{a}$						
Muslim	0.150 (0.0126)	0.045 (0.3395)	0.338 ($<0.0001$)	0.040 (0.2867)	0.147 (0.0007)	0.110 (0.0162)
Christian	0.452 ($<0.0001$)	0.0089 (0.8439)	0.497 ($<0.0001$)	-0.008 (0.8197)	0.342 ($<0.0001$)	0.024 (0.5811)
Wealth						
Poor$^\mathrm{a}$						
Middle	0.179 ($<0.0001$)	-0.008 (0.575)	0.222 ($<0.0001$)	-0.053 ($<0.0001$)	0.182 ($<0.0001$)	-0.039 (0.0002)
Rich	0.570 ($<0.0001$)	-0.075 ($<0.0001$)	0.467 ($<0.0001$)	-0.114 ($<0.0001$)	0.418 ($<0.0001$)	-0.110 ($<0.0001$)
Marital status						
Unmarried$^\mathrm{a}$						
Married/cohabited	-0.055 ($<0.0001$)	2.53 ($<0.0001$)	-0.040 ($<0.0001$)	2.76 ($<0.0001$)	-0.018 (0.0314)	1.98 ($<0.0001$)
Separated/other	0.070 (0.0002)	2.40 ($<0.0001$)	0.060 ($<0.0001$)	2.60 ($<0.0001$)	0.007 (0.4921)	1.91 ($<0.0001$)
Occupation						
Not working$^\mathrm{a}$						
Domestic/informal	-0.031 (0.0023)	0.030 (0.0099)	-0.007 (0.2929)	0.032 (0.0007)	-0.0004 (0.9523)	0.038 ($<0.0001$)
Professional/formal	0.118 ($<0.0001$)	0.009 (0.6331)	0.504 ($<0.0001$)	-0.205 ($<0.0001$)	0.333 ($<0.0001$)	-0.084 ($<0.0001$)
Contraceptive use						
Non-user/other$^\mathrm{a}$						
User	0.139 ($<0.0001$)	0.175 ($<0.0001$)	0.074 ($<0.0001$)	0.151 ($<0.0001$)	0.039 ($<0.0001$)	0.179 ($<0.0001$)
Place of residence						
Urban$^\mathrm{a}$						
Rural	-0.245 ($<0.0001$)	0.128 ($<0.0001$)	-0.185 ($<0.0001$)	0.133 ($<0.0001$)	-0.184 ($<0.0001$)	0.146 ($<0.0001$)
Region						
Northern$^\mathrm{a}$						
Central	-0.167 ($<0.0001$)	0.022 (0.2718)	-0.180 ($<0.0001$)	0.020 (0.1345)	-0.088 ($<0.0001$)	-0.024 (0.0678)
Southern	-0.152 ($<0.0001$)	-0.035 (0.079)	-0.182 ($<0.0001$)	-0.009 (0.5097)	-0.104 ($<0.0001$)	-0.016 (0.2263)
Current age	-0.027 ($<0.0001$)	0.058 ($<0.0001$)	-0.024 ($<0.0001$)	0.056 ($<0.0001$)	-0.020 ($<0.0001$)	0.059 ($<0.0001$)
Age at 1st sex	0.027 ($<0.0001$)	-0.025 ($<0.0001$)	0.028 ($<0.0001$)	-0.033 ($<0.0001$)	0.037 ($<0.0001$)	-0.041 ($<0.0001$)
Correlation (pval)	-0.623 ($<0.0001$)		-0.676 ($<0.0001$)		-0.617 ($<0.0001$)	
AIC	99,862.18		190,917.78		201,588.40	

$^\mathrm{a}$ = reference category, pv = *p*-value

After removing the outliers using a cutoff of $\pm 1.96$ of the deviance residual, there was a significant improvement in the model’s AIC and *p*-value estimates compared to the original models (see Table 6). Additionally, there was a substantial increase in the effects of ethnicity, place of residence, household wealth, and religion on the schooling outcome. Similarly, the effects of marital status and the use of modern contraceptive methods on schooling decreased significantly. The effect of age at first sex on both schooling and fertility remained unchanged even after the removal of the outliers from the model. On the other hand, there was a marked increase in the effects of household wealth and marital status on fertility. Religion, region, and ethnicity remained insignificant on fertility. The correlation estimates also slightly decreased in the three data sets. These results suggest that the removal of outlier women from the data improved the model fit.

**Table 6:** Effects of women socio-demographic characteristics on years of schooling and fertility outcomes upon fitting bivariate Poisson model to the MDHS data sets without outlier observations beyond cutoff $\pm 1.96$ of deviance residual

	2004MDHS ($\boldsymbol{n=11,369}$)		2010 MDHS ($\boldsymbol{n=22,571}$)		2015-16 MDHS ($\boldsymbol{n=24,072}$)	
	Schooling	Fertility	Schooling	Fertility	Schooling	Fertility
Variable	Log-Mean (pv)	Log-Mean (pv)	Log-Mean (pv)	Log-Mean (pv)	Log-Mean (pv)	Log-Mean (pv)
Intercept	1.41 ($<0.0001$)	-2.92 ($<0.0001$)	1.47 ($<0.0001$)	-2.92 ($<0.0001$)	1.40 ($<0.0001$)	-2.27 ($<0.0001$)
Ethnicity						
Tumbuka/Tonga/ot$^\mathrm{a}$						
Lomwe/Yao/Sena	-0.135 ($<0.0001$)	0.007 (0.6639)	-0.094 ($<0.0001$)	-0.001 (0.9081)	-0.055 ($<0.0001$)	-0.007 (0.6071)
Chewa/Nyanja	-0.097 ($<0.0001$)	0.041 (0.0108)	-0.106 ($<0.0001$)	0.022 (0.0462)	-0.087 ($<0.0001$)	0.019 (0.0903)
Religion						
None/Other$^\mathrm{a}$						
Muslim	0.174 (0.005)	0.056 (0.2422)	0.335 ($<0.0001$)	0.027 (0.4650)	0.143 (0.0012)	0.103 (0.0260)
Christian	0.481 ($<0.0001$)	0.021 (0.6490)	0.489 ($<0.0001$)	-0.022 (0.5389)	0.338 ($<0.0001$)	0.016 (0.7232)
Wealth						
Poor$^\mathrm{a}$						
Middle	0.185 ($<0.0001$)	-0.008 (0.5780)	0.225 ($<0.0001$)	-0.049 ($<0.0001$)	0.183 ($<0.0001$)	-0.039 (0.0003)
Rich	0.577 ($<0.0001$)	-0.078 ($<0.0001$)	0.467 ($<0.0001$)	-0.110 ($<0.0001$)	0.418 ($<0.0001$)	-0.111 ($<0.0001$)
Marital status						
Unmarried$^\mathrm{a}$						
Married/cohabited	-0.039 (0.0026)	2.60 ($<0.0001$)	-0.029 (0.0009)	2.86 ($<0.0001$)	-0.014 (0.0735)	2.06 ($<0.0001$)
Separated/other	0.090 ($<0.0001$)	2.47 ($<0.0001$)	0.071 ($<0.0001$)	2.71 ($<0.0001$)	0.012 (0.2622)	1.98 ($<0.0001$)
Occupation						
Not working$^\mathrm{a}$						
Domestic/informal	-0.032 (0.0017)	0.030 (0.0106)	-0.008 (0.2256)	0.033 (0.0006)	0.001 (0.8047)	0.035 (0.0001)
Professional/formal	0.116 ($<0.0001$)	0.011 (0.5262)	0.500 ($<0.0001$)	-0.209 ($<0.0001$)	0.328 ($<0.0001$)	-0.091 ($<0.0001$)
Contraceptive use						
Non-user/other$^\mathrm{a}$						
User	0.128 ($<0.0001$)	0.157 ($<0.0001$)	0.065 ($<0.0001$)	0.135 ($<0.0001$)	0.036 ($<0.0001$)	0.171 ($<0.0001$)
Place of residence						
Urban$^\mathrm{a}$						
Rural	-0.244 ($<0.0001$)	0.131 ($<0.0001$)	-0.183 ($<0.0001$)	0.140 ($<0.0001$)	-0.180 ($<0.0001$)	0.152 ($<0.0001$)
Region						
Northern$^\mathrm{a}$						
Central	-0.157 ($<0.0001$)	0.038 (0.0661)	-0.173 ($<0.0001$)	0.026 (0.0563)	-0.082 ($<0.0001$)	-0.017 (0.1952)
Southern	-0.138 ($<0.0001$)	-0.025 (0.2159)	-0.174 ($<0.0001$)	0.006 (0.6523)	-0.099 ($<0.0001$)	-0.006 (0.6286)
Current age	-0.027 ($<0.0001$)	0.059 ($<0.0001$)	-0.024 ($<0.0001$)	0.057 ($<0.0001$)	-0.020 ($<0.0001$)	0.060 ($<0.0001$)
Age at 1st sex	0.027 ($<0.0001$)	-0.025 ($<0.0001$)	0.028 ($<0.0001$)	-0.033 ($<0.0001$)	0.037 ($<0.0001$)	-0.042 ($<0.0001$)
Correlation (pval)	-0.624 ($<0.0001$)		-0.679 ($<0.0001$)		-0.613 ($<0.0001$)	
AIC	96,167.60		185,901.16		195,464.44	

$^\mathrm{a}$ = reference category, *pv=* *p*-value

**Fig. 4:**
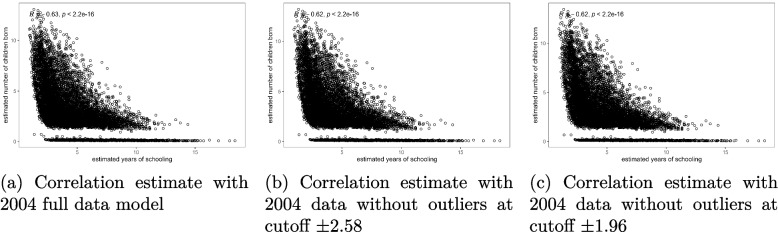
Correlation between female education and fertility before and after removing outliers from the bivariate Poisson model, 2004 MDHS data. Source: Researcher

**Fig. 5:**
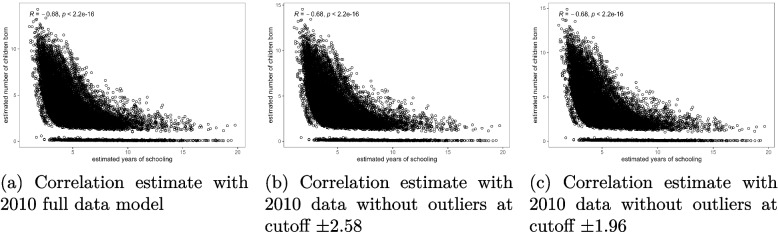
Correlation between female education and fertility before and after removing outliers from the bivariate Poisson model, 2010 MDHS data. Source: Researcher

**Fig. 6:**
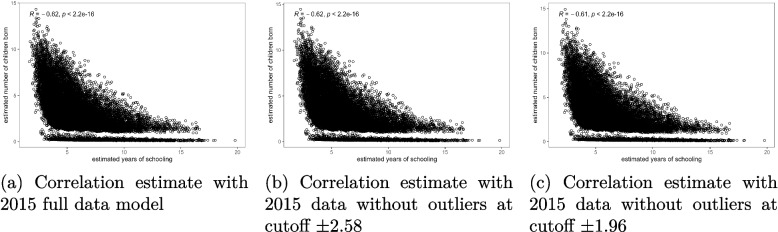
Correlation between female education and fertility before and after removing outliers from the bivariate Poisson model, 2015 MDHS data. Source: Researcher

The results presented in Figs. 4, 5 and 6 show that there was no significant change in the correlation between female schooling and fertility after removing the outlier observations from the model. The slopes of the scatter plots in Figs. 4(a), 5(a), and 6(a) were similar to those in Figs. 4(b-c), 5(b-c), and 6(b-c). All the graphs confirmed a negative correlation between female education and fertility. The re-estimated Spearman correlation coefficient values overlaid on Figs. 4(b-c), 5(b-c), and 6(b-c) after removing outliers from the analysis indicated that the correlation between female schooling and fertility in Malawi ranged from -0.68 to -0.61 during the period of analysis. These estimates were significantly different from zero and approximately double the raw estimates given in Tables 1, 2 and 3. Additionally, Figs. 4(a) through 6(c) showed that while there was a general negative linear relationship between female schooling and fertility, the strength of the relationship was not the same for all schooling years. Regardless of the status of outliers in the model, the slope of the fertility curves was steeper for the lower number of schooling years up to 5 years, gentle between 5 and 10 years, and became flatter as education duration increased beyond 10 years.

## Discussion

This article explored the relationship between female education and fertility rates in Malawi. It specifically investigated the impact of outlier women on this relationship using a bivariate Poisson regression model. The majority of women in the study had attended between 1 and 8 years of schooling, and had given birth to 1 to 4 children. This trend is consistent with previous studies, which show that Malawi’s high-quality programmes aimed at reducing unwanted pregnancies have been successful [2, 14, 23]. The study found that the correlation between fertility and women’s education in Malawi remained steady, ranging from -0.68 to -0.61 throughout the period of observation. This means that women with more years of schooling tended to have fewer children and vice versa. This correlation is attributed to the delay or acceleration in maternal age that schooling induces [5, 47, 59, 77]. However, the study observed that this relationship was not uniformly linear for all years of schooling. It was strongest with a steeper slope up to five years of schooling, followed by a gentler slope between five and ten years of mother education. The linear association was weaker with a flatter slope for female education beyond ten years. This explains why the correlation between the two variables is weaker in developed nations, where most women have attended more than ten years of schooling, compared to developing countries where there are mixed groups of low- and highly-educated women [35, 36, 47, 57].

The study conducted diagnostic analyses and found some unusual cases of women in the bivariate Poisson model. These outliers were women who either had no education or had completed at least nine years of education and had either no children or at least five children. Most of the outliers did not use modern contraceptive methods, were domestic workers, or had non-formal employment. Previous research has shown that side effects and social norms are the main reasons why modern contraceptive methods are not used in rural Malawi [16, 62]. However, the general uptake of modern contraceptives at the national level is high, with more than half of the adult female population using them [25]. This could explain why non-users of modern contraceptives were identified as outliers in this study. They belonged to a population that had generally adopted family planning methods. Domestic workers in Malawi are known to face various human rights abuses, including being denied the right to education [58]. Therefore, it is not surprising that some of the detected outliers in this study were domestic workers with no schooling. Some of the observed outliers who had no children and were not using modern contraceptives might be school-going adolescents aged 15-19 years who lack knowledge about and access to contraceptives [19, 53].

The study found that the presence of outliers in the model had a noticeable impact on the model estimates, depending on the depth of cutoff used for the diagnostic statistic. When using the cutoff with a larger error rate on the distribution of the residual, substantial changes were observed in the ML estimates. However, the changes in correlation estimates were minimal regardless of the choice of the cutoff for the residual. This suggests that the inclusion of outlying women in the bivariate Poisson model biased the ML estimates more than the correlation coefficient. Further analysis showed that the detected outliers had a similar correlation structure for female education and fertility as the well-fitted observations. This could explain why they had less impact on the overall correlation, as is the case with other statistical measures when the data are missing at random [24]. The influence of each observation on the regression parameter estimates is the product of its outlier values and leverage in the fitted model. When the observations are dropped as a group in the model, their influence on the parameter estimates is usually compounded [40, 79]. This could be the reason why the maximum likelihood estimates were impacted more than the correlation, when the outliers were removed from the analysis in this study. To improve the fit of the model to data and provide assurance to the researcher in the findings and conclusions being made, it is desirable to deal with outlier observations in the modelling process. When the goal of the study is to improve the fit of the model to data, robust estimation techniques can be used to improve the model estimates and predictions [17]. These methods are known to be less affected by outliers in the model and produce lower standard errors compared to maximum likelihood [28, 52].

The study found that a woman’s marital status, occupation, place of residence, contraceptive use, current age, household wealth, and age at first marriage were significantly associated with both her education and fertility. On the other hand, her religion, ethnicity, and region only affected her education and not her fertility. The study also found that Muslim and Christian women had significantly higher levels of education compared to those with no religion. Additionally, women from middle to rich households, those who got separated or divorced, those with professional and formal occupations, those who used modern contraceptives, and those with increased age at first sex had significantly higher levels of education. The study also found that women from the *Lomwe*, *Yao*, *Sena*, *Chewa*, and *Nyanja* ethnic groups had shorter schooling durations compared to those from *Tumbuka*, *Tonga*, *Ngoni* and other related tribes. Married women, those with domestic and non-formal occupations, those from rural locations, those from central and southern regions, and those with higher current age also had shorter schooling durations. The low education attainment in non-religious communities in Malawi may be due to delayed primary school enrollment and high drop-out rates due to low motivation from family members in such populations [60]. Meanwhile, early marriages are probably the main cause of the observed short duration of schooling in females of *Lomwe*, *Yao*, *Sena*, *Chewa* and *Nyanja* ethnic origins [9]. Whereas contraceptive usage and professional occupation are the by-products of knowledge acquisition, which is why these factors are associated with a higher number of schooling years in females [34]. The low education attainment in married women could be due to early marriages that cut the education journey faster than expected or may reflect the division of labor within the home, where women attend to most household chores in developing nations and have less time to study, as well as maternity breaks from school to take care of pregnancy [22, 30].

It has been observed that there is a significant increase in fertility in married and separated/divorced women compared to unmarried women. Women with domestic and non-formal occupations were found to have higher fertility rates than those who were not working. Similarly, women from rural areas have a higher fertility rate than those from urban settings. Furthermore, modern contraceptive users tend to have higher fertility rates than non-users, and older women have higher fertility rates than younger women. On the other hand, fertility outcomes were significantly lower in women from middle and rich households compared to poor households. Women with professional and formal occupations had lower fertility rates than non-working women, and women with a higher age at first sexual intercourse also had lower fertility rates. These results are consistent with findings from other studies. For example, the low fertility rate in professional women and those with higher age at first sex is attributed to delayed maternal age [3, 73]. Also, previous studies have observed an increased fertility rate in women who use modern contraceptive methods in Malawi [21].

## Conclusion

This study aimed to investigate the effect of outlier women on the correlation between female education and fertility in Malawi. The study analysed three demographic and health survey data sets and used a bivariate Poisson regression model. Outliers were identified as women who had either no education or at least nine years of schooling and had either no children or at least five children, which was not typical for most women. Most of these outlier women did not use modern contraceptive methods and worked as domestic or non-formal employment workers. The study revealed a high negative correlation between female education and fertility in Malawi from 2000 to 2016, ranging from -0.68 to -0.61. The correlation was stronger for women with up to five years of education and weaker beyond ten years. When the outliers were removed from the analysis, their influence was more substantial on regression coefficient estimates than on the correlation estimate.

The majority of the women studied had attended between one to eight years of school and had given birth to one to four children. Muslim and Christian women, wealthier families, divorced or separated women, those in professional and formal occupations, users of modern contraceptive methods, and older women were found to have a higher number of years of schooling. On the other hand, those in the *Lomwe*, *Yao*, *Sena*, *Chewa* and *Nyanja* ethnic groups, married women, domestic and non-formal job servants, rural residents, those who lived in the central and southern regions, and older women had a shorter duration of schooling. Moreover, the fertility rate was high in married women, domestic and non-formal occupation workers, users of modern contraceptive methods, rural residents, and older women, while the rate was low in wealthier females, those in professional and formal occupation servants, and women who had first sex at an older age. There was no association between region of stay, religion, or ethnic group and a woman’s fertility.

This study suggests using the bivariate Poisson regression approach to analyse the relationship between female education and fertility. This method considers socio-cultural factors and any outliers in the data. Policymakers in education and health should initiate programmes to enhance women’s education levels and reproductive health, particularly for domestic workers. Health policymakers in Malawi must assess the efficacy of modern contraceptive methods in reducing the fertility rate as they currently contribute to the high fertility rate. However, due to the large number of zero values in both the schooling and fertility data, future research could explore this association using zero-truncated bivariate Poisson or bivariate negative binomial regression methods. Notably, the R package VGAMdata, which was used to fit the bivariate Poisson model in this study, does not provide estimates for the covariance between the two count response variables being analysed nor process most of the residuals. Therefore, in this study, it was post-estimated separately using purposefully coded R programmes. The study recommends embedding these post-estimation statistics into the VGAMdata package for future research.

### Supplementary Information

**Additional file 1.**

## Data Availability

The 2004, 2010, and 2015-16 MDHS data are publicly and freely available for users at https://dhsprogram.com/data/available-datasets.cfm.
