# Supplementary material for: A bivariate Poisson regression to analyse impact of outlier women on correlation between female schooling and fertility in Malawi
Source: BMC Womens Health. 2024 Jan 20;24:55. doi: 10.1186/s12905-024-02891-w (PMC10799448; doi:10.1186/s12905-024-02891-w)
Supplement: Supplementary file 1 — Additional file 1. [file 12905_2024_2891_MOESM1_ESM.pdf]

1 Appendix 1: STATA code used for data cleaning

```
#STATA code used for data cleaning
codebook v024
gen region = v024
codebook v025
gen residence = v025
codebook v130
replace v130=1 if v130==99
gen religion=.
replace religion = 1 if v130==7
replace religion = 1 if v130==96
replace religion = 2 if v130==6
replace religion = 3 if v130==5
replace religion = 3 if v130==4
replace religion = 3 if v130==3
replace religion = 3 if v130==2
replace religion = 3 if v130==1
codebook v131
replace v131=1 if v131==99
gen ethnicity=.
replace ethnicity=1 if v131==13
replace ethnicity=1 if v131==96
replace ethnicity=1 if v131==2
replace ethnicity=1 if v131==4
replace ethnicity=1 if v131==7
replace ethnicity=1 if v131==8
replace ethnicity=1 if v131==10
replace ethnicity=1 if v131==11
replace ethnicity=2 if v131==3
replace ethnicity=2 if v131==5
replace ethnicity=2 if v131==6
replace ethnicity=3 if v131==1
replace ethnicity=3 if v131==9
replace ethnicity=3 if v131==12
codebook v190
gen wealth=.
replace wealth=1 if v190==1
replace wealth=1 if v190==2
replace wealth=2 if v190==3
replace wealth=3 if v190==4
replace wealth=3 if v190==5
gen current_age=v012
gen marital_status=.
replace marital_status=1 if v501==0
replace marital_status=2 if v501==1
replace marital_status=2 if v501==2
replace marital_status=3 if v501==3
replace marital_status=3 if v501==4
replace marital_status=3 if v501==5
gen schooling=v133
gen fertility=v201
```

```
codebook v364
gen modern_contraceptive=.
replace modern_contraceptive=1 if v364==4
replace modern_contraceptive=1 if v364==3
replace modern_contraceptive=1 if v364==2
replace modern_contraceptive=2 if v364==1
codebook v717
gen occupation=.
replace occupation=1 if v717==0
replace occupation=2 if v717==3
replace occupation=2 if v717==4
replace occupation=2 if v717==6
replace occupation=2 if v717==9
replace occupation=2 if v717==8
replace occupation=3 if v717==1
replace occupation=3 if v717==2
replace occupation=3 if v717==7
replace occupation=3 if v717==5
gen age_at_1st_sex = v531
replace age_at_1st_sex =rpoisson(16.59018) if age_at_1st_sex >=50
replace age_at_1st_sex =rpoisson(16.59018) if age_at_1st_sex <=5
hist age_at_1st_sex
gen schooling_grpd=.
replace schooling_grpd=1 if schooling==0
replace schooling_grpd=2 if schooling==1
replace schooling_grpd=2 if schooling==2
replace schooling_grpd=2 if schooling==3
replace schooling_grpd=2 if schooling==4
replace schooling_grpd=2 if schooling==5
replace schooling_grpd=2 if schooling==6
replace schooling_grpd=2 if schooling==7
replace schooling_grpd=2 if schooling==8
replace schooling_grpd=3 if schooling > 8
gen fertility_grpd=.
replace fertility_grpd=1 if fertility==0
replace fertility_grpd=2 if fertility==1
replace fertility_grpd=2 if fertility==2
replace fertility_grpd=2 if fertility==3
replace fertility_grpd=2 if fertility==4
replace fertility_grpd=3 if fertility>=5
gen region2=.
replace region2=1 if region==2
replace region2=0 if region2==.
gen region3=.
replace region3=1 if region==3
replace region3=0 if region3==.
gen residence2=.
replace residence2=1 if residence==2
replace residence2=0 if residence2==.
gen ethnicity2=.
replace ethnicity2=1 if ethnicity==2
replace ethnicity2=0 if ethnicity2==.
```

```
gen ethnicity3=.
replace ethnicity3=1 if ethnicity==3
replace ethnicity3=0 if ethnicity3==.
gen wealth2=.
replace wealth2=1 if wealth==2
replace wealth2=0 if wealth2==.
gen wealth3=.
replace wealth3=1 if wealth==3
replace wealth3=0 if wealth3==.
gen maritalstatus2=.
replace maritalstatus2=1 if marital_status==2
replace maritalstatus2=0 if maritalstatus2==.
gen maritalstatus3=.
replace maritalstatus3=1 if marital_status==3
replace maritalstatus3=0 if maritalstatus3==.
gen contraceptive2=.
replace contraceptive2=1 if modern_contraceptive==2
replace contraceptive2=0 if contraceptive2==.
gen occupation2=.
replace occupation2=1 if occupation==2
replace occupation2=0 if occupation2==.
gen occupation3=.
replace occupation3=1 if occupation==3
replace occupation3=0 if occupation3==.
gen religion2=.
replace religion2=1 if religion==2
replace religion2=0 if religion2==.
gen religion3=.
replace religion3=1 if religion==3
replace religion3=0 if religion3==.
```

2 Appendix 2: R code used for bivariate Poisson model fitting, outlier and correlation analyses

```
#R code for bivariate Poisson model fitting, outlier and correlation
    ↪ analyses

rm(list=ls())
library(car)
library(foreign)
library(VGAMdata)
library(ggplot2)
library(reshape2)
library(nnet)
library(ggrepel)
library(dplyr)
library(data.table)
library(readstata13)
library(ggpubr)
require(foreign)
```

```
mydata = read.dta("C:/Users/User/Documents/rdata2015.dta",convert.
    ↪ factors=F)

model1 <- vglm(cbind(schooling, fertility) ~ religion2 +religion3 +
    ↪ region2 + region3+residence2+ethnicity2+ethnicity3+wealth2+
    ↪ wealth3+current_age+maritalstatus2+maritalstatus3+
    ↪ contraceptive2+occupation2+occupation3+age_at_1st_sex,
    ↪ poissonff, data = mydata)

summary(model1)

AIC = -2*-50279.06 + 2*34

cor.test(mydata$schooling,mydata$fertility,method="spearman")

mydata$theta_sch = exp(coef(model1)[1]+mydata$religion2*(coef(model1
    ↪ )[3]) +mydata$religion3*(coef(model1)[5]) +mydata$region2*(
    ↪ coef(model1)[7]) + mydata$region3*(coef(model1)[9])+mydata$
    ↪ residence2*(coef(model1)[11])+mydata$ethnicity2*(coef(model1)
    ↪ [13])+mydata$ethnicity3*(coef(model1)[15])+mydata$wealth2*(
    ↪ coef(model1)[17])+mydata$wealth3*(coef(model1)[19])+mydata$
    ↪ current_age*(coef(model1)[21])+mydata$maritalstatus2*(coef(
    ↪ model1)[23])+mydata$maritalstatus3*(coef(model1)[25])+mydata$
    ↪ contraceptive2*(coef(model1)[27])+mydata$occupation2*(coef(
    ↪ model1)[29])+mydata$occupation3*(coef(model1)[31])+mydata$age
    ↪ _at_1st_sex*(coef(model1)[33]))

mydata$theta_tfr = exp(coef(model1)[2]+mydata$religion2*(coef(model1
    ↪ )[4]) +mydata$religion3*(coef(model1)[6]) +mydata$region2*(
    ↪ coef(model1)[8]) + mydata$region3*(coef(model1)[10])+mydata$
    ↪ residence2*(coef(model1)[12])+mydata$ethnicity2*(coef(model1)
    ↪ [14])+mydata$ethnicity3*(coef(model1)[16])+mydata$wealth2*(
    ↪ coef(model1)[18])+mydata$wealth3*(coef(model1)[20])+mydata$
    ↪ current_age*(coef(model1)[22])+mydata$maritalstatus2*(coef(
    ↪ model1)[24])+mydata$maritalstatus3*(coef(model1)[26])+mydata$
    ↪ contraceptive2*(coef(model1)[28])+mydata$occupation2*(coef(
    ↪ model1)[30])+mydata$occupation3*(coef(model1)[32])+mydata$age
    ↪ _at_1st_sex*(coef(model1)[34]))

cor.test(mydata$theta_sch,mydata$theta_tfr,method="spearman")

corr1 <- ggplot(mydata,aes(x=mydata$theta_sch,y=mydata$theta_tfr))+
    ↪ geom_point(pch=1)+stat_cor(method="spearman")+labs(x="
    ↪ estimated␣years␣of␣schooling",y="estimated␣number␣of␣children
    ↪ ␣born")+theme_test()

ggsave('test.tiff', corr1, device = "tiff", units="in", width=6,
    ↪ height=4, pointsize=12,bg="white", dpi = 1200)

mydata$r1 = mydata$schooling - mydata$theta_sch
```

```r
mydata$r2 = mydata$fertility - mydata$theta_tfr

mydata$sgn1=ifelse(mydata$r1 > 0,1,-1)

mydata$sgn2=ifelse(mydata$r2 > 0,1,-1)

mydata$d1=mydata$sgn1*sqrt(2*(mydata$schooling*log((mydata$schooling +0.000001)/mydata$theta_sch)-mydata$r1))

mydata$d2=mydata$sgn2*sqrt(2*(mydata$fertility*log((mydata$fertility +0.000001)/mydata$theta_tfr)-mydata$r2))

mydata$Di = (mydata$d1 + mydata$d2)/2

mydata$id = 1:nrow(mydata)

outlierII04 <- ggplot(mydata,aes(x=mydata$id,y=mydata$Di))+geom_point(pch=1)+labs(x="woman id",y="outlier estimates to bivariate Poisson model")+geom_text(aes(label = mydata$id), size = 2.8, vjust = 0.5)+ theme_test()+geom_hline(yintercept = c(-2.58,2.58),color=c('red','red'))

ggsave('test.tiff', outlierII04, device = "tiff", units="in", width=6, height=4.5, pointsize=12,bg="white", dpi = 1200)

write.table(mydata,file="newMWIR2004.csv",sep=",",row.names = F)
```
